# A Review on Rolling Bearing Fault Signal Detection Methods Based on Different Sensors

**DOI:** 10.3390/s22218330

**Published:** 2022-10-30

**Authors:** Guoguo Wu, Tanyi Yan, Guolai Yang, Hongqiang Chai, Chuanchuan Cao

**Affiliations:** 1 College of Energy and Power Engineering, Lanzhou University of Technology, Lanzhou 730050, China; 2 School of Intelligent Manufacturing Engineering, Chongqing University of Arts and Sciences, Chongqing 402160, China

**Keywords:** rolling bearing, fault detection, type of fault signal, diagnostic method, sensor

## Abstract

As a precision mechanical component to reduce friction between components, the rolling bearing is widely used in many fields because of its slight friction loss, strong bearing capacity, high precision, low power consumption, and high mechanical efficiency. This paper reviews several excellent kinds of study and their relevance to the fault detection of rolling bearings. We summarize the fault location, sensor types, bearing fault types, and fault signal analysis of rolling bearings. The fault signal types are divided into one-dimensional and two-dimensional images, which account for 40.14% and 31.69%, respectively, and their classification is clarified and discussed. We counted the proportions of various methods in the references cited in this paper. Among them, the method of one-dimensional signal detection with external sensors accounted for 3.52%, the method of one-dimensional signal detection with internal sensors accounted for 36.62%, and the method of two-dimensional signal detection with external sensors accounted for 19.72%. The method of two-dimensional signal detection with internal sensors accounted for 11.97%. Among these methods, the highest detection rate is 100%, and the lowest detection rate is more than 70%. The similarities between the different methods are compared. The research results summarized in this paper show that with the progress of the times, a variety of new and better research methods have emerged, which have sped up the detection and diagnosis of rolling bearing faults. For example, the technology using artificial intelligence is still developing rapidly, such as artificial neural networks, convolutional neural networks, and machine learning. Although there are still defects, such methods can quickly discover a fault and its cause, enrich the database, and accumulate experience. More and more advanced techniques are applied in this field, and the detection method has better robustness and superiority.

## 1. Introduction

With economic development and scientific and technological progress, rolling bearings are increasingly used in mechanical devices. Researchers have conducted various theoretical and practical research on them [1], and the related research technology is gradually becoming mature. Therefore, summarizing the detection methods of rolling bearing fault signals is of far-reaching significance and influence.

A rolling bearing is a precision and essential mechanical component that transforms the sliding friction between the shaft and shaft seat into rolling friction and reduces friction loss. It comprises four parts: an inner ring, an outer ring, a rolling body, and a cage. The role of the inner ring is to coordinate and rotate with the axis. The outer ring matches the bearing seat and plays a supporting role. Thus, there is relative movement between the inner and outer rings [2]. The cage distributes the rolling body evenly between the inner and outer rings. The shape, size, and quantity of the rolling elements directly affect the performance and life of the rolling bearing. The cage can not only distribute the rolling body evenly, but also lubricate the rolling body to guide its rotation.

Bearings are widely used in various fields of the economy [3]. In the national economy, they are mainly involved in agriculture [4], mining [5], manufacturing [6], electricity [7], heat [8], water production [9] and construction [10], transportation, postal services [11] and many other industries. They are used in automobiles [12], agricultural machinery, industrial production (machining tools) [13], drills in mining, textile machinery in the manufacturing industry, cranes in the construction industry, all kinds of transmissions, and so on [14]. Furthermore, rolling bearings can be used in various environmental conditions, such as high winds; high and low temperatures; strong magnetic fields; and in acidic, alkali, and other media. Regarding the bearing industry, being the foundation and pillar of the machinery industry, its development level often represents or restricts the development level of a country’s machinery industry and other related industries. In a body, bearings are often important or core parts.

The main faults on rolling bearings are on the inner and outer ring surfaces, raceway surface, rolling body surface, rolling body and raceway contact surface, cage and rolling body or seat ring surface, ring edge and roller end face, and inner and outer ring raceway surface and subsurface [15,16,17]. At the same time, different bearing elements have corresponding defect frequencies, inner ring defect frequency (BPFI), outer ring defect frequency (BFPO), ball bearing defect frequency (BSF), and cage defect frequency (FTF). The calculation formulae of the four defect frequencies are as follows:

(1) $$\mathrm{BPFI}=\frac{Nb\;S}{2}\left(1+\left(\frac{Bd}{Pd}\right)\cos A\right)$$

(2) $$\mathrm{BPFO}=\frac{Nb\;S}{2}\left(1-\left(\frac{Bd}{Pd}\right)\cos A\right)$$

(3) $$\mathrm{BSF}=\frac{Pd\;S}{Bd}\left(1-{\left(\frac{Bd}{Pd}\right)}^{2}\cos^{2}A\right)$$

(4) $$\mathrm{FTF}=\frac{\;S}{Bd}\left(1-\left(\frac{Bd}{Pd}\right)\cos A\right)$$

*Nb* represents the number of balls, *S* represents speed, *Bd* represents ball diameter, *Pd* represents Pitch diameter, and *A* represents the contact angle (degrees).

This review mainly discusses two significant aspects of one-dimensional signal analysis and two-dimensional image analysis. Among them, in terms of sensor detection of faults, learning from a single sensor is extended to learning from multiple sensors. The multi-frequency information fusion method of sensors divides the multi-frequency information and fuses the information of different frequencies to achieve fault diagnosis (Cang Liu et al., 2022) [18]. There is also a novel approach to signal analysis in one dimension, which adjusts the optimal parameters of the adaptive function, the improved grey wolf optimized variational modal decomposition (IGVMD), and adjusts the non-linear convergence factor adjustment strategy, the grey wolf adaptive position update strategy, and the Levy flight strategy in the IGWO algorithm (Xiaofeng Wang et al., 2020) [19]. The recent research on two-dimensional images includes the MTF-ResNet fault diagnosis model, which combines the Markov transition field and a residual network. A residual network is used to extract features from the vibration signal conversion graph more thoroughly and effectively through the Markov transition field (Jialin Yan et al., 2022) [20].

**Figure 1:**
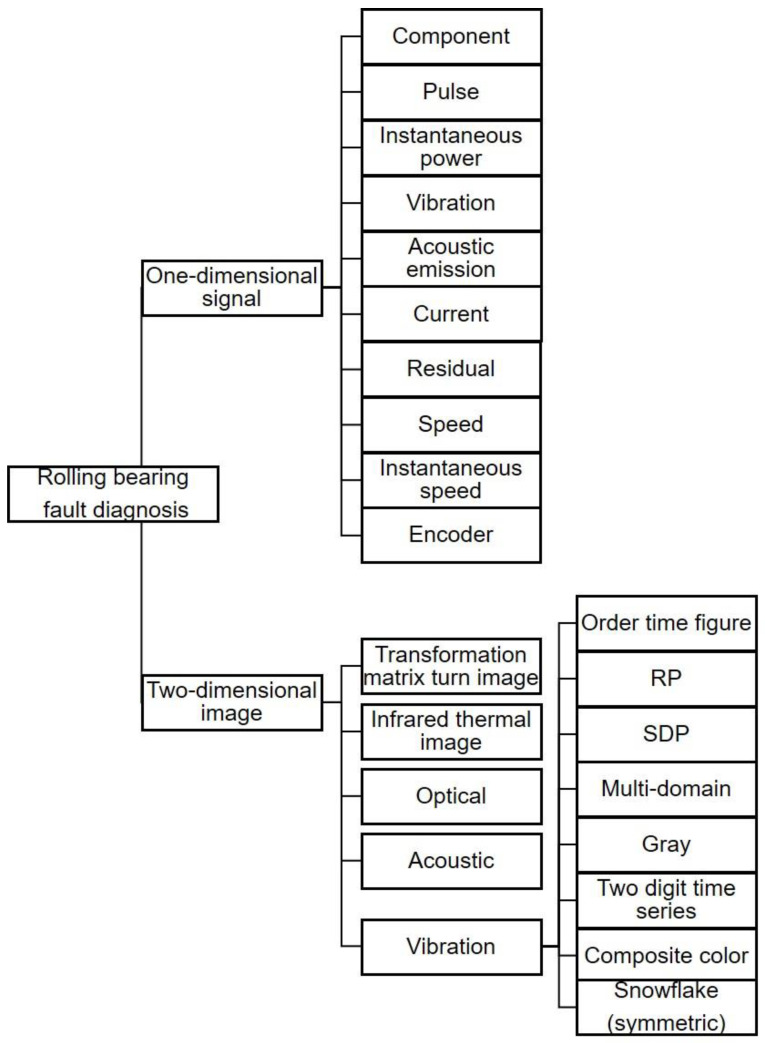
Diagram of the main content of this review.

In order to let beginners quickly start in this field, we have summarized the detection methods of different sensors to obtain rolling bearing fault signals (Figure 1).

## 2. Research on the Faults of Rolling Bearings

### 2.1. The Fault Locations in or on the Rolling Bearing

**Figure 2:**
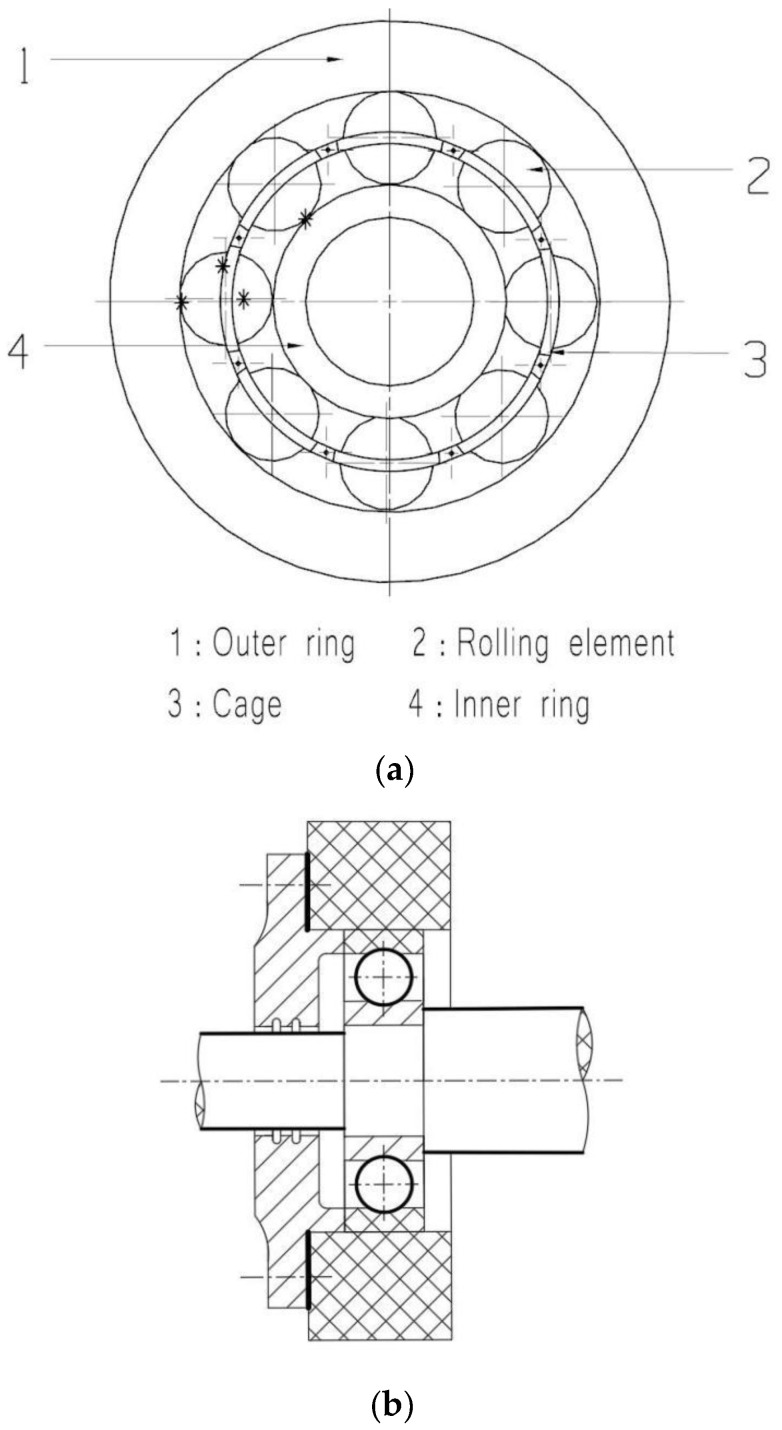
We take deep-groove ball rolling bearings as an example. (**a**) Rolling bearing plane view, where * marks the fault location. Taking deep-groove ball rolling bearings as an example. (**b**) Lateral section view of rolling bearing and shaft in assembly.

According to the different fault parts of rolling bearings, the faults of rolling bearings are classified as outer ring faults, inner ring faults, rolling body faults, and cage faults [21,22,23]. Such fault classification according to the components of rolling bearings has the advantages of being intuitive and concise. The specific fault location is marked in Figure 2a; see Table 1 for details.

### 2.2. Types of Rolling Bearing Failure

Of course, there are also fault classification for specific fault conditions, which are fatigue peeling, wear, abrasion, pitting, creep, rust, electrical corrosion, plastic deformation, indentation, scratch, strain, adhesion, burn, glue, fracturing, cage damage, scratch card injury, hydrogen embrittlement, etc. The general fault types on the working surface are wear, abrasion, pitting, creep, rust, electrical corrosion, plastic deformation, indentation, collision, strain, adhesion, burn, etc. [22]. Here, the bearing’s working surface refers to the outer ring, inner ring, raceway, and surface of the rolling body [21,23]. Generally, glue and cage damage appear in the rolling body and raceway contact surface. Generally, faults on the ringside and roller end face are scratches. Hydrogen embrittlement is generally the fault type on the surfaces and subsurfaces of the inner and outer raceway. If the rolling bearing has defects in the material, or is exposed to an improper level of heat in the process of machine operation, or if there is a heavy load, a fracture will occur. The assembly of the rolling bearing and shaft is shown in Figure 2b.

### 2.3. Types of Sensor

According to the research and analysis of rolling bearing faults in the past ten years, we conclude that the installation positions of sensors for detecting rolling bearing faults can be classified as external and internal. Sensors installed externally for fault detection mainly include sensors for vibration signal detection, that is, vibration sensors [24]; sensors that detect temperature signals—temperature sensors [25]; sensors used to detect acoustic emission signals—acoustic emission sensors [26]. In addition, the acquired images are analyzed and diagnosed, including vibration images, acoustic images, spectral images, and infrared thermal images [27]. Sensors installed in an internal system mainly include sensors that detect current signals, such as eddy current sensors [28]; and sensors that detect rotational speed, speed, neutral point voltage, instantaneous power, instantaneous speed, residual signal, pulse signal, component signal, and encoder signal [29]. Sensors for detecting magnetic field frequency, such as optical sensors for extracting magnetic field frequency features, are others [30]. One can obtain the time-frequency image signal, obtain the original signal, and transform the matrix into an image, etc. [31]. There is a kind of optical fiber sensor that can be installed both internally and externally [32].

Similarly, signal acquisition by sensors and detection of rolling bearing faults can be divided into one-dimensional signal detection and two-dimensional signal detection according to the different dimensions of the detection objects. These two types can be further divided into signal and image signal sensors. The detection method of a one-dimensional signal sensor is to analyze and diagnose the signal detected by the sensor directly. The sensors used to detect one-dimensional signals are mainly divided into a few categories. In vibration sensors, the parameters of the detected vibration signals are analyzed, mainly the air gap function. Such sensors can also detect sparse resonant signals. Acoustic emission sensors test the electrical parameters of the sensor and the electrical parameters of the primary current signal. It is typical to perform an analysis of load torque, rotational speed signal, speed signal, magnetic field frequency, neutral point voltage, instantaneous power, transient rotation speed, residual signal, pulse signal, and component signals, such as the encoder’s signal [33]. The detection method of a two-dimensional signal system is to carry out mathematical calculations or build a model or neural network to transform the signal(s) detected by the sensor(s) into two-dimensional images and then analyze and diagnose them. The two-dimensional signals are mainly vibration images, including gray images, two-dimensional time series images, composite color images, snowflake images (symmetric images), multi-domain images, etc. Other methods include acoustic imaging, spectral imaging, time-frequency image, SDP imaging (using the symmetric lattice principle), infrared thermal imaging, recursive plot (RP) analysis, and using a transformation matrix to image [34]. Regarding what has been discussed above, see Table 1 for details.

## 3. Detection Methods Based on One-Dimensional Signals

The researchers analyze the detected signals directly. Most of the one-dimensional signals analyzed are vibration signals, including sparse resonant signals. Others include temperature signals, acoustic emission signals, current signals, speed signals, magnetic field frequency, neutral voltage, instantaneous power, instantaneous speed, residual signal, pulse signal, component signal, encoder signal, and other signals with detection characteristics [24,28,35,36,37].

### 3.1. Signals Detected by External Sensors and Their Analysis Methods

According to previous studies, most vibration signals are detected by external sensors.

Vibration signals often accompany rolling bearing faults. After sensors obtain vibration signals, methods are needed to analyze or filter them if they are noisy. For example, the fast Fourier transform (FFT) and continuous wavelet transform (CWT) are used to perform envelope detection analysis of vibration signals with low signal-to-noise ratios, and those showing pulse loads caused by defects, in which case the bearing is established as a spring-mass-damping system model (Deepak et al., 2014) [38]. The high-frequency details of the denoised second-generation wavelet signal can be decomposed into several generation functions (PFs) by spline LMD. Power spectrum analysis can then be applied to the power spectrum to detect bearing fault information and identify fault modes (Wen, C.-Y et al., 2015) [39]. The vibration signal obtained in a noisy environment was directly input into a CNN detection method (Hoang D.-t et al., 2019) [40]. Noise is removed by matching the vibration texture mode with a wave atomic basis function. Artificial neural networks are used to detect and identify unique fault features generated by different parts of a machine (Jha et al., 2020) [41]. As researchers improve their methods, we can see how diagnostics are evolving.

Sparse signal decomposition of the resonance of vibration signals with the optimal mass factor (Q factor) was performed after processing, and the low-resonance components were analyzed by combining empirical mode decomposition and energy operator demodulation (Yan Lu, 2019) [42]. There are also methods to extract features from the original vibrations for diagnosis. Instantaneous rotational speed (IRS) was input to a novel knowledge transfer network with a sparse autoencoder and deep convolutional neural network (KTN-SAEDCNN) (Chen, Peng et al., 2020) [43]. These methods of studying and analyzing vibration signals and their related parameters are becoming increasingly intelligent. The methods and other factors mentioned in this paragraph are shown in Table 2.

### 3.2. Signal Detected by an Internal Sensor and Analysis Methods

Current signal detection and feature analysis are the most common and widely used methods in one-dimensional signal analysis.

There are many kinds of current signal analysis methods, and they are commonly used. In many pieces of literature, researchers have summarized several classical methods. They common ones are the demodulation and transformation of the signal, the use of artificial intelligence for extensive data analysis, time-frequency analysis of the non-stationary signal generated in the running of the bearing, spectrum analysis of the harmonic extraction of the complex component of the signal, and a real-time online remote monitoring method. These methods represent different analytical perspectives and can lead to different experimental conclusions.

#### 3.2.1. The Demodulation Transformation Signal Analysis Method

Since the current signal is modulated by phase angle and amplitude, one could use a signal analysis method to understand modulation transformation. For example, the Teager–Kaiser energy operator demodulated the stator current to reveal faults (Pineda-Sanchez et al., 2013) [44]. Through induction motor stator current analysis (MCSA), the modulated signal bispectrum (MSB) was used to detect and diagnose various motor bearing defects (Ahmed Alwodai et al., 2013) [45]. The Xiang Gong et al. (2013) bearing fault diagnosis of a variable-speed direct-drive wind turbine based on single-phase stator current measurement was carried out by using an appropriate current frequency and amplitude demodulation algorithm and a 1P constant power spectral density algorithm [46] based on the phase modulation theory of BLODT; that is, the stator current was derived from the Bessel function theory to study the current characteristics and diagnose faults. A test rig was established to simulate single-point defects by generating torque vibration for verification (Xinbo Wu et al., 2015) [47]. A modulated signal bispectral (MSB) detector was used to identify the spectral components of fundamental and characteristic frequencies to detect rolling bearing faults (Xi Chen et al., 2020) [48]. The methods of current signal detection mentioned in this section are detailed in Table 3.

#### 3.2.2. Artificial Intelligence Signal Analysis Methods

Signal processing using machine learning techniques and unsupervised classification techniques such as artificial ant clustering has been used for fault diagnosis (Soualhi, A et al., 2013) [49]. High-resolution, wideband, synchronous resampling of current signals was performed with a novel algorithm for efficient computation, and faults were detected by identifying excitation from the spectrum of synchronous sampled stator current signals using a pulse detection algorithm (Gong, Xiang et al., 2015) [50]. Similarly, a support vector machine (SVM) can be used to classify and diagnose different types of bearing faults (Shrinathan et al., 2019) [51]. In one study, through motor CS deep learning and information fusion (IF), signal features were extracted, and then they introduced the decision-level IF technology of a convolutional neural network (CNN) for classification and eventual fault diagnosis (Hoang et al., 2020) [52]. Fault diagnosis using a new DL architecture built by deep-sincnet was reported by Abid, Firas Ben, et al. [53].

**Figure 3:**
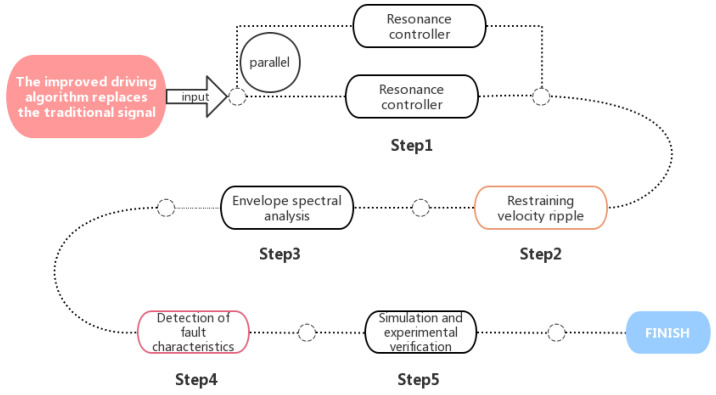
Diagram of a process where noise is eliminated by a driving algorithm before signal acquisition.

**Figure 4:**
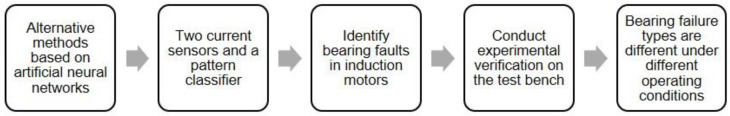
The method of combining information theory measurement and intelligent tool detection.

In the signal-based, adaptive, semi-supervised framework C-ASSF, a Wasserstein generative adversarial network (WGAN-GP) with gradient punishment was used to extract only recognizable features from standard current signals. Line spectrum feature extraction (LSFE) technology removed the current signal’s primary frequency component. Finally, an indicator indicating the degree of deviation from a normal distribution was used to identify external bearing faults in the transmission system (Jie Li et al., 2021) [54]. In another study, a novel program was developed to automatically diagnose different types of bearing faults and load anomalies through stator current and external stray magnetic flux measured in bearing-mounted induction motors (Marcello et al., 2021) [55]. Other researchers used a driving algorithm to eliminate noise components before data acquisition. The resonant controller was set in parallel with the proportional integral controller to suppress the velocity fluctuations. The envelope spectrum analysis method was used to detect the fault characteristics (Ming Yang et al., 2020) [56]. This method is illustrated in Figure 3. A comprehensive study of information theory measurement and intelligent tool fault detection led to a time-domain fault-feature-extraction method based on the mutual information between two phase current signals (Tiago et al., 2020) [57]. The flow of this method is shown in Figure 4.

The above methods can be seen in Table 4.

#### 3.2.3. Time Frequency Analysis Signal Analysis Methods

Time-frequency analysis is a common fault detection method. This method can provide good rolling bearing fault diagnosis efficiency and is convenient and fast. However, the only critical point and difficulty is the applicability and debugging of the established model and parameters to the actual situation. Time-frequency analysis methods, including time-domain analysis and frequency-domain analysis, are common. Good results have been achieved in theoretical research and practical applications. Researchers usually analyze the current spectrum and frequency spectrum. For example, integrated empirical mode decomposition (EEMD) is used for rolling bearing fault detection in stationary and non-stationary situations (Amirat Y et al., 2012) [58]. Stator current spectrum subtraction and related residual energy are used as diagnostic indicators to carry out fault diagnosis as well (ElBouchikhi, E et al., 2013) [59]. A method exists for spectral estimation of fault frequencies via maximum likelihood estimator (MLE) (El Bouchikhi et al., 2015) [60]. Other authors presented a spectrum synchronization (SS) technique using the current signal for early IM defect detection (Li, De Z. et al., 2015) [61]. In addition, by analyzing the square envelope spectrum of stator current, experimental bearing fault detection of a three-phase induction motor was carried out (Leite et al., 2015) [62].

In one study, features were evaluated by linear discriminant analysis, and fault diagnosis was performed using A Bayesian classifier (Mboo et al., 2016) [63]. In another study, spectrum analysis of the effect of slot-order harmonics on stator current was used to detect bearing failure (Song, XJ et al., 2017) [64]. A current noise elimination method using time shift for spectral analysis was presented by Dalvand, Fardin, et al. [65]. Fault features are extracted from stator currents by continuous wavelet transform (CWT) for fault diagnosis (Singh et al., 2017) [66]. There is a method of current noise elimination and fault diagnosis that uses linear prediction theory and optimal prediction order (Dalvand, Fardin et al., 2018) [67].

Here is a brief description of the formula of a continuous wavelet transform (CWT). Let f (t) be a square-integrable function, denoted as:

(5) $$f\left(t\right)\in L^{2}\left(R\right)$$

ψ (t) is a wavelet function. Then:

(6) $$W_{f}\left(a,b\right)=\frac{1}{\surd a}\int_{-\infty }^{+\infty }f\left(t\right)\varphi^{*}\left(\frac{t-b}{a}\right)\mathrm{dt}=<f(t),\varphi_{a,b}\left(t\right)>\;$$

f (t) is called the wavelet transform. a is the scale factor (a > 0); b is the time shift factor, which can be positive or negative; and ψ* () indicates ψ*. The sign <x, y> represents the inner product, defined as:

(7) $$\langle x\left(t\right),y\left(t\right)\rangle =\int^{\;}x\left(t\right)y^{*}\left(t\right)\mathrm{dt}$$

The displacement and scaling of wavelet functions, where a, b, and t are continuous variables:

(8) $$\varphi_{a,b}(t)=\frac{1}{\sqrt{a}}\varphi \left(\frac{t-b}{a}\right)$$

By comparing and analyzing different fault classification parameters with motor current characteristic analysis (MCSA)-obtained parameters, fault diagnosis was performed by Kompella et al. [68]. In another study, the instantaneous frequency (IF) of IM stator current was analyzed by Hilbert transform, and the frequency related to bearing outer-raceway faults was detected by fast Fourier transform (FFT) spectrum analysis (Song, XJ et al., 2018) [69]. In another study, a hybrid bfd method based on optimized static wavelet packet transform for feature extraction and fault classification of artificial immune systems nested within support vector machines was presented (Ben Abid, ETAL., 2018) [70].

In one study, the stator current of the defective induction motor rolling bearing (IMRB) was modeled according to the equivalent magnetic circuit (MEC). Then, the induction motor (IM) was modeled by MEC, and the magnetic equivalent network was formed by connecting the flux tube and node. They solved the current stator model. Finite element analysis and dynamic testing were performed on a typical IM to verify. They thus conducted fault diagnosis (Han, Qinkai et al., 2019) [71].

Combined with current analysis and deep learning, the classification methods of wavelet packet transform and the deep one-dimensional convolutional neural network containing a softmax layer were used separately to extract features of stator current signals for fault diagnosis (Kao, IH et al., 2019) [72].

New indicators extracted from electrical signals were fed into an adaptive neural fuzzy inference system (ANFIS) for fault diagnosis (Soualhi, Moncef et al., 2019) [73]. In another study, an induction motor (IM)-related, electromechanical magnetic coupling model based on MWFA was used to extract the harmonic component and amplitude of a fault excitation in the stator current. Then a new evaluation index, FHD (fault excitation harmonic distortion), was used to describe the specific relationship between the fault size and the severity of fault excitation harmonic distortion (Wang, Chen et al., 2021) [74]. Fault diagnosis by analyzing current characteristics (Victor Avina-Corral et al., 2021) [75].

As opposed to recursive plot (RP) analysis of the two-dimensional signal, there is a quantitative method of detecting the characteristics of ball bearings by vibration spectrum analysis of a one-dimensional signal: recursive quantification analysis (QRA). Based on the vibration of the self-aligning ball bearing at different clearance levels and speeds, the vibration spectrum was obtained, and the bearing characteristics were obtained (Bartłomiej Ambrożkiewicz et al., 2022) [76]. When this method is used in the detection of bearing faults, it has good comparative data and can determine the fault locations and causes through data analysis.

The methods discussed in this subsection are detailed in Table 5.

#### 3.2.4. Spectral Analysis Signal Analysis Methods

Let us go through some examples—first, single-point defect detection of bearings based on parameter spectral estimation. Based on bearing fault characteristic frequency, multidimensional MUSIC (MD MUSIC) was developed for bearing fault detection (Elbouchikhi et al., 2016) [77]. One can also use high-resolution spectral analysis technology to obtain the fault’s sensitive frequency, to obtain its corresponding amplitude for fault diagnosis (Trachi, Y et al., 2016) [78]. There is also current-characteristic analysis for three-phase induction motor bearing fault detection. Under various conditions, the current is analyzed in the frequency domain, and high-resolution techniques, such as the matrix pencil method and wavelet denoising, are used to evaluate the fault degree (K. C. Deekshit Kompella et al., 2020) [79]. There is a method to detect vibration signals by installing an acceleration sensor used for state detection inside a bearing and decomposing the detected acceleration signals into angular and linear components. The analyzed data can be used for bearing fault diagnosis (Vladimir V Sinitsin et al., 2017) [80]. This has reference value. The comparison of the two methods is shown in Table 6.

#### 3.2.5. Remote Real-Time Monitoring Methods

Remote real-time monitoring and online diagnosis can diagnose faults in real-time, but it takes a long time and costs a lot. The following are some examples. A remote monitoring method for radial load deep groove ball bearings in LBE based on torque is presented in [81]. In another study, the features of stator current extraction were analyzed, and the feature knowledge database for online fault detection was established (Yang, T et al., 2016) [82]. The in-line status monitoring technique was used to analyze the stator-current fault components of the tested induction motor for identification and fault severity in the study by Corne, B et al. [83]. A new, multi-candidate, low-delay detection algorithm for online detection of various possible bearing faults has been reported [84]. However, if real-time monitoring is combined with an algorithm, the number of possible operations of the machine will be reduced.

For the treatment of air gap function, the air gap function can be processed in a multi-coupling circuit model to obtain an extensive harmonic list of the stator-current spectrum regarding bearing faults, and then frequency analysis is carried out to achieve fault diagnosis (Ojaghi, M. et al., 2018) [85].

Table 7 summarizes the methods discussed in this section.

Current signal analysis is universal.

**Figure 5:**
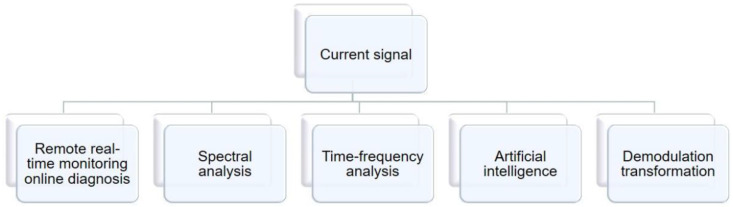
One-dimensional current-signal analysis methods.

The classification of current signal methods is shown in Figure 5.

#### 3.2.6. Other Methods Based on One-Dimensional Signals

As it is challenging to extract acoustic emission signals from rolling bearing faults, there are few methods to study the signals. The samples’ entropy and the Lempel–Ziv complexity of the morphological pattern spectrum (MPS) curve of collected acoustic emission signals were examined by a multi-scale morphological analysis program to diagnose bearing health (Wen-Jing et al., 2016) [86]. This is one of the typical methods for the characteristic analysis of acoustic signals.

The collection process of speed signals can be regarded as the speed measurement process of rotating machinery. Under the conditions of variable speed and constant load, the BFD (RSB-BFD) method based on rotor speed was created (Hamadache et al., 2015) [87]. It can extract and analyze the rotor speed and study the motor speed. Based on motor-speed-kurtosis spectrum analysis, a diagnostic method which improves the signal-to-noise ratio (SNR) was presented (Boyang et al., 2019) [88]. The speed signal is one of the indispensable signals in mechanical motion and has high research value.

The velocity signal is one of the most fundamental signals of moving objects. By analyzing the primary frequency component, characteristics of the velocity signal and residual signal can be obtained. Research methods include, for example, signal analysis of a brushless dc (BLDC) motor and fault diagnosis through the effects on hall sensors (Omid Zandi et al., 2019) [89]. Diagnostic methods using sensor data are extremely important and influential.

As one of the fundamental indexes of power quality, magnetic field frequency is an important parameter to show the running state of rolling bearings. Fault diagnosis of rolling bearings has been performed using magnetic-field characteristic-frequency expressions derived from multiple modulations (Tianyu Geng et al., 2016) [90]. The researchers analyzed not only the frequencies of the spectrum, but also the magnetic field frequencies.

The neutral point voltage was analyzed using a new comprehensive modeling and analysis method for a three-degrees-of-freedom induction motor and an effective bearing loosening characteristic method (Mohammad J. Jafarian et al., 2020) [91].

The potential difference is one of the characteristic parameters. Only a few methods use it, but with high reliability.

The instantaneous power has been studied, and a motor-bearing-fault identification system was developed which employs the commonly used motor-stator current and voltage (Irfan, M et al., 2015) [92].

The research and analysis of pulse signals are based on adaptive algorithms that function according to peak energy processing of pulse signal features captured by “W” structural elements, parallel-frequency-domain spectral correlation analysis, and fault identification according to correlation coefficients (Qiang et al., 2021) [93]. These methods analyze the frequency spectrum of periodic discrete signals to detect faults.

One can study the component signals that carry information. The envelope spectrum can be obtained by analyzing the component signals converted into a one-dimensional sampling time series, and then fault diagnosis can be carried out (Leng et al., 2014) [94].

The signal converted by the encoder of a high-precision device was studied. A framework for health assessment of rotating machinery using rotary encoders was also presented. Finally, an adaptive denoising method based on the Gini coefficient was used for fault diagnosis (Zhao, Ming et al., 2018) [95].

Table 8 summarized this section below for more clarity.

## 4. Detection Methods Based on Two-Dimensional Signals

**Figure 6:**
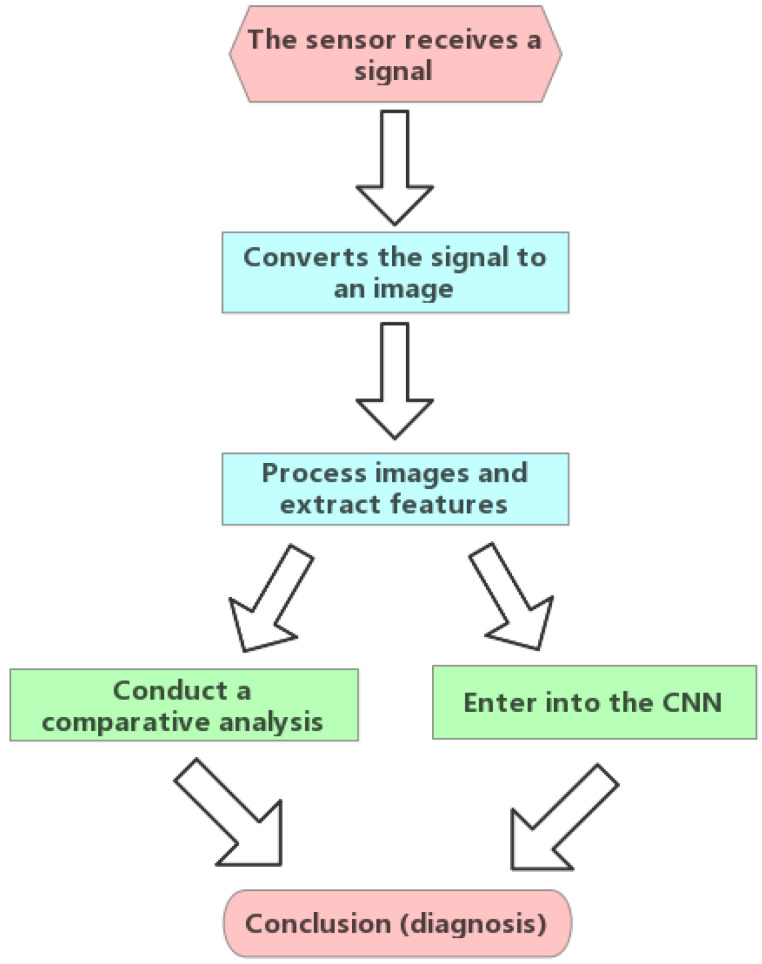
Flow chart of two-dimensional signal method summary.

The researchers converted images of the detected signals and analyzed the resulting images. Mainly vibration images, including gray image, two-dimensional time series image, composite color image, snowflake image, multi-domain image, SDP image, recursive plot (RP) analysis and order graph, etc. Acoustic image, spectral image, time-frequency image, infrared thermal image and transformation matrix image are also popular images for research. Summary of the process of two-dimensional signal method. (Figure 6) The main methods of fault diagnosis through two-dimensional signals are described in the following sections.

### 4.1. Images Obtained by External Sensor Detection and Analysis Methods

#### 4.1.1. Methods Based on Vibration Images

**Figure 7:**
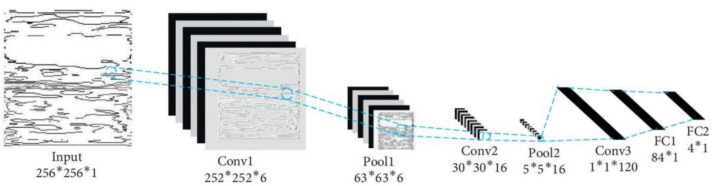
CNN structural diagram [99].

There have been two main strategies in vibration image research in recent years. One is to extract features and compare them. The second is to input the converted image into a CNN for analysis. Take the following examples. A pre-processed short-time Fourier transform (STFT) was used to appropriately adapt an image classification transformer (ICT) for rolling bearing fault detection (Christos T. Alexakos et al., 2021) [96]. Image features were extracted, model functions were established, and data and functions in different states were compared to realize fault diagnosis (Bin Liu et al., 2021) [97]. Analysis of vibration signals, the establishment of images with the persistent spectrum, comparison between images, bearing fault detection, and diagnosis under fixed and time-varying speed conditions were performed by Mohamed et al. [98]. Vibration images with four different features generated by the original signal were tested by a CNN (Fan, Hongwei et al., 2021) [99]. The Da-rnn model was used to convert vibration signals into images and then input them to the embedded CBAM convolutional neural network (CNN) model for fault classification (Jun Li et al., 2022) [100]. A graphic-based WGAN with GP data extension and CNN verification was presented (Hongwei Fan et al., 2022) [101]. In another study, the images generated by the gram angle field (GAF) were input to DenseNet, and DenseNet performed feature extraction of two-dimensional images for rolling bearing fault diagnosis (JIANG Jiaguo et al., 2021) [102]. Finally, in other research, the vibration signals were converted into 2D images and input into an improved CNN model for fault diagnosis (School of Mechanical and Electrical Engineering, 2021) [103]. It can be seen that vibration images are widely used in the analysis of two-dimensional signals. Since a CNN model is used most often, its general structure is displayed in Figure 7. The comparison of methods using vibration images is summarized in Table 9.

#### 4.1.2. Grayscale Image Methods

Generally, the input one-dimensional vibration signal is converted to a two-dimensional grayscale vibration image and then to an RGB vibration image (RGBVI). The automatic learning of RGBVIs features via CNN has been used to classify bearing health status (Hosameldin O. A. Ahmed et al., 2021) [104]. The features of images converted from extracted signals can be learned by a multi-class support vector machine (MSVM) to diagnose rolling bearing faults (Jha, Rakesh Kumar et al., 2021) [105]. In one study, off-line convolutional neural networks (OFF-CNN) and online convolutional neural networks (ON-CNN) with the same model structure were constructed using multi-channel data fusion and gray image transformation input.

A rolling bearing fault diagnosis model based on off-CNN obtained source-domain features and model parameters in the whole connection layer and initialized on-CNN parameters (Quansheng Xu et al., 2022) [106]. The state detection and fault identification of rolling bearings were performed on gray scale images by a GAN using SECNN (Hongtao Tang et al., 2021) [107]. Vibration signals were converted into grayscale images and convolved with different convolution kernels to realize an MCNN and data fusion model constructed by multi-scale sample input for fault diagnosis (Lv, Defeng et al., 2021) [108]. See Table 10 for a summary of these methods.

#### 4.1.3. SDP Image Methods

Consider the following examples. The incremental cumulative holographic symmetric lattice (SDP) feature fusion method is a visual diagnosis method that converts feature signals into SDP images (Xuewei Song et al., 2022) [109]. The SDP can be combined with a snowflake image: the vibration signal is converted into a symmetric image that is then input into the optimal convolutional neural network (CNN) model for fault diagnosis using the symmetric lattice (SDP) principle (Yongjian Sun et al., 2022) [110]. In another study, SDP images were used for analysis. Bearing fault diagnosis was based on improved Chebyshev distance, IMF1, in SDP images and on empirical mode decomposition (EMD) of vibration signals (Sun, Yongjian, 2021) [111]. The specific methods are compared in Table 11.

#### 4.1.4. Other Types of Vibration Image Methods

A fault reconstruction feature classification method for subway-train axle box rolling bearings was based on a multi-scale stacked feeling field of a deep residual network (Yu, Hu et al., 2022) [112].

Composite color images can be used for analysis. An MTL-based CNN architecture was input into fused multi-domain information to construct composite color images for fault diagnosis (Hasan et al., 2022) [113].

Snowflake images can be analyzed. Empirical mode decomposition (EMD) is used to transform vibration signals into eigenmode functions in snowflake images to extract (IMF) components for symmetric-lattice-image fault diagnosis (Sun, Yongjian, 2021) [114]. A CNN can convert vibration signals made by SDP into visual snowflake images via a softmax classifier to conduct a CNN comparison and detection of rolling bearing fault signals with an SDP image sample library in various states (Wang, H, 2019) [115].

Research on multi-domain images also exists. For example, DNN’s multi-branched structure was used to process multi-domain images for feature extraction for fault diagnosis (Van-Cuong Nguyen et al., 2021) [116].

Analysis was performed by recursive plot (RP) analysis. A human vision system (HVS) was used to extract fault features after vibration signal transformation for recursive plot (RP) analysis for fault diagnosis (Yujie Cheng et al., 2017) [117].

The order graph can be analyzed. An identification sequence diagram of the rolling body bearing defect was used in a diagnostic method for variable conditions (speed and load) through a CNN. (Tayyab et al., 2022) [118]. In order to reduce the interference of uncertainties and other factors, a Markov transition field (MTF) and convolutional neural network (CNN) were automatically combined after learning data features. The CNN extracts original time series and converts them into feature information in images, which is a diagnosis method with high accuracy and can be used for fault classification (Mengjiao Wang et al., 2022) [119]. See Table 12 for the comparison of these methods.

#### 4.1.5. Methods Based on Other Two-Dimensional Images

Regarding the study and analysis of acoustic images, the CNN recognition wave stacking method (WSM) can be used for acoustic imaging fault detection via the non-contact ABD method (Ran Wang et al., 2020) [120].

A CNN classifier can be used to identify spectral images transformed from time-domain vibration signals to diagnose rolling bearing faults (Youcef Khodja et al., 2020) [121]. Another fault diagnosis method is based on signal conversion into a moment-invariant S-transform and image Hu processing; that is, using the bearing signal and time-frequency spectrum to create two-dimensional images (Guo, JF et al., 2013) [122]. Deep CNN can use transfer learning to train with bispectrum images of fault signals for fault diagnosis (Chhaya Grover et al., 2022) [123].

In infrared thermal image research and analysis, CNNs and ANNs are used to classify and compare non-invasive thermal images of rolling bearings in different states for fault diagnosis (Choudhary et al., 2021) [124].

See Table 13 for the comparison of these methods.

### 4.2. Analysis Methods of Images Detected by Internal Sensors

#### 4.2.1. Time-Frequency Image-Based Methods

For the study and analysis of time-frequency images, the threshold determined by the time-frequency image transformation of vibration signals can be processed by binarization of a mask template, and the principal component with higher energy in the signal is then reserved for fault diagnosis (Ma, Yunchao et al., 2021) [125]. Alternatively, one can use the clustering centers extracted from time-frequency images converted from vibration signals by the EMD-PWVD method to realize fault identification (Hongwei Fan et al., 2020) [126]. The signal can be converted to a time-frequency image by the replacement of the last fully connected layer of the universal Alexnet bearing fault diagnosis model (Wang, JY et al., 2019) [127]. Sparse TFI texture features were extracted by input signal processing in a classifier for rolling bearing fault diagnosis (Du, Y et al., 2018) [128]. Fault diagnosis has also been based on wavelet time-frequency image analysis, feature texture extraction, and label consistency K-SVD classification (Yuan, HD et al., 2018) [129].

The deep learning featureless method automatically learns the signal images generated by feature data analysis for fault classification and diagnoses faults (David Verstraete et al., 2017) [130]. The SPWVD time-frequency analysis method, Wigner–Ville distribution time-frequency analysis method, and wavelet scale spectrum method were used to obtain texture feature vectors from time-frequency distribution images. The SPWVD method had the highest classification accuracy and sensitivity (Wang, Yaping et al., 2017) [131]. A singular-value decomposition-dimension-reduction method was used to suppress background noise in original time-frequency images and diagnose bearing faults (Wang, Y et al., 2016) [132]. Support vector machines were used to identify smooth pseudo-Wigner–Ville distribution (SPWVD) feature vectors obtained by position-constrained linear coding (LLC) and the spatial pyramid method to diagnose bearing faults (Wei Gang Wang et al., 2014) [133]. The Hough transform was used to analyze the Wigner–Weil time-frequency distribution for fault diagnosis by processing images (Li, Hongkun et al., 2010) [134].

A simple speed-free sequential tracking method based on the visualized sequential spectrum obtained from signal processing was used for fault diagnosis (Wang, Y et al., 2019) [135]. Texture features were extracted from time-frequency images transformed from processed vibration signals to diagnose bearing faults (Yang Hongbai et al., 2017) [136]. Sensor-based bearing vibration signals can be used to construct feature data sets to solve fault diagnosis by mode classification and recognition(Zhao, MB et al., 2014) [137]. Sliding global gradient class activation mapping (SGG-CAM) was used to analyze the anti-noise capability of a module, and time-frequency images were input into the EMSCNN network based on the MS-D module and RCA module for fault diagnosis (Hongchun Sun et al., 2022) [138]. Fault detection using multi-sensory data fusion of time-frequency images into a deep residual network was performed (Gultekin, Ozgur et al., 2022) [139]. Time-frequency image samples were processed by deep convolutional generation and continuous wavelet transform. The diversity of generated images was evaluated by the image quality evaluation metrics of SSIN and PSNR, considering the classification of network expansion faults. Then, a convolutional neural network classified the extended time-frequency image data set to achieve fault diagnosis (Tian Han et al., 2021) [140]. The summary is shown in the Table 14.

#### 4.2.2. Converting the Transformation Matrix to an Image

Images transformed by a transformation matrix were studied and analyzed, and the PCA transformation matrix was obtained from the samples, which were then input into the convolutional neural network (CNN) model for fault diagnosis through the expansion dimension of cascade spatial projection (CSP) (Yunji Zhao et al., 2022) [141].

**Figure 8:**
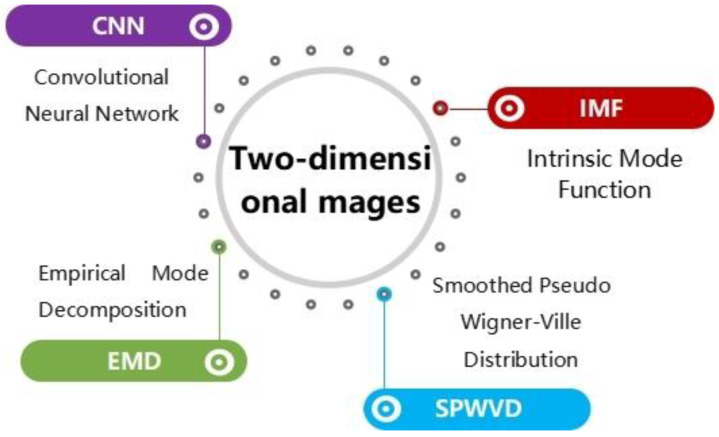
Several common methods of diagnosing bearing faults with two-dimensional images.

In summary, 2D image analysis methods for diagnosing bearing faults mainly include convolutional neural network extraction and recognition, IMF analysis, empirical mode decomposition, SPWVD, and so on. That is how you analyze a two-dimensional image (Figure 8).

## 5. Summary of Problems and Prospects for the Future

This paper is a summary of fault diagnosis methods of rolling bearings that involve sensors. The rolling bearing’s structure, fault location, and fault type have been shown and listed. According to different locations of faults, there are different appropriate sensors. Then, we listed the methods that use the two kinds of signals (one- and two-dimensional), focusing on the comparison between them, and then summarized the research methods used in the past ten years. We now put forward the shortcomings of the existing methods and prospects for industry research.

Systems have overlapping signal processing methods, such as Fourier transformation, whether they process one-dimensional or two-dimensional signal information. In a noisy environment, noise reduction is also carried out to extract the fault signal more accurately. We compared all the methods and found that the non-real-time methods worked best. The methods seeing the most use were the analysis of vibration signals, images, and current signals.

We believe that the influential factors of rolling bearing fault research are as follows:

(1) Artificial intelligence diagnosis based on data fusion.

We found that these methods require large amounts of data and many calculations, thereby requiring a large amount of running memory to run. They also produce errors and cannot find all the faults that have been experimented with.

(2) Applicability of bearing types.

By comparing the above methods, we found that some fault diagnosis methods are only suitable for a specific type of bearing, not for all bearings. This requires us to continue studying and improving bearing diagnosis methods’ applicability.

(3) Continuing to optimize and improve the methods which have already been proposed.

In summarizing the methods, we found that some are optimized or improved versions of already existing diagnostic methods. This aspect is precious and meaningful, and it will promote the development of bearing fault diagnosis methods. We believe that this still needs to be pursued.

(4) Reducing and solving the interference related to the technology used and the environment by data processing.

Most of the noise problems and the corresponding solutions are in the methods themselves. We perform filtering in order to obtain accurate signals. There is bound to be missed noise though.

(5) Optimization and upgrading of sensor research.

At present, additional sensors need to be optimized to make it more convenient to sense and collect fault signals to diagnose rolling bearings. Correspondingly, improving the internal sensors’ accuracy is also imperative.

In summarizing the methods, we found that image sensors are seldom used for rolling fault bearing diagnosis. Researchers should be bold and innovative. The CCD and CMOS of image sensors can be applied to image recognition of bearing fault diagnosis. Attention should also be paid to using basic sensors in the detection systems. The development and use of multiple sensors will also be significant. We should also pay attention to simplification and innovation in research.

There are many methods for analyzing signals. By comparing the research results with each other, it can be found that as time goes on, the diagnostic methods being developed become more robust, effective, accurate, and superior in general, and can be combined with artificial intelligence algorithms. Research on rolling bearing fault detection and diagnosis is improving, and more and more methods are becoming available. With the development of science and technology, detection methods in the future will be more intelligent, efficient, and convenient. These efforts will also require many scientific researchers to continue improving the rolling bearing fault detection and diagnosis methods.

## Figures and Tables

**Table 1:** A list of internal and external sensors with one-dimensional and two-dimensional signals.

Sensor	External Sensor	Internal Sensor
**A one-dimensional signal**	Vibration signal	
	Acoustic emission signal	Current signal
		Speed signal
		Rotate speed signal
		The magnetic field frequency
		Neutral voltage
		Instantaneous power
		The instantaneous speed
		The residual signal
		The pulse signal
		Component of the signal
		Encoder signal
**A two-dimensional signal**	Vibration image	The time-frequency image
	Gray image	The transformation matrix transforms the image
	Two-dimensional time series images	
	Composite color image	
	Snowflake image (symmetric image)	
	Multi-domain image	
	SDP image	
	recursive plot (RP) analysis	
	Order time figure	
	The acoustic image	
	Spectral image	
	Infrared thermal image	

**Table 2:** Summary and comparison table of vibration signal analysis methods.

Purpose	Applied Environment	Fault Signal	Fault Types	Signal Processing Method	Based Model or Theory	Reference
The vibration signals generated by bearing defects were analyzed under low SNR.	Low signal-to-noise ratio conditions, pulsed loads caused by defects	Vibration signal	Defective bearing in inner raceway and rolling body	Envelope Detection, Fast Fourier Transform (FFT) and Continuous Wavelet Transform (CWT)	The bearing is modeled as a spring-mass-damping system	Deepak et al., 2014 [38]
In order to extract the characteristic fault frequency of a weak bearing signal	In the strong noise		Inner and outer ring fault	Second generation wavelet denoising, strip LMD decomposition		Wen, C.-Y et al., 2015 [39]
Improve the performance of intelligent fault diagnosis methods	Noise environment			Convolutional neural network	CNN model	Hoang D.-T. et al., 2019 [40]
Rolling bearing fault diagnosis	Different load and noise conditions			Matching of vibration texture mode and wave atomic basis function	ANN	Jha et al., 2020 [41]
The periodic impact component is extracted from the multi-component mixed vibration signal effectively.	In the presence of multi-component mixed vibration signals			Genetic algorithm, resonant sparse signal decomposition, empirical mode decomposition and energy operator demodulation are combined		Yan Lu, 2019 [42]
Fault detection of rolling bearing				Deep convolutional neural network (DCNN)	Knowledge transfer model of KTN-SAEDCNN	Chen, Peng et al., 2020 [43]

**Table 3:** Summary and comparison table of demodulation transformation methods.

Purpose	Fault Signal	Fault Types	Signal Processing Method	Based Model or Theory	Reference
Rolling bearing fault detection and diagnosis	Current signal		Teager–Kaiser energy operator		Pineda-Sanchez et al., 2013 [44]
		Baseline, outer ring failure and inner ring failure	Modulated signal bispectrum (MSB)		Ahmed Alwodai et al., 2013 [45]
			The method consists of appropriate current frequency and amplitude demodulation algorithms and a 1P constant power spectral density algorithm		Xiang Gong et al., 2013 [46]
		Electric corrosion	Modulated signal bispectral (MSB) detector	Overlapping segmentation	Xi Chen et al., 2020 [48]
Determine the severity of rolling bearing failure			The form of stator current is deduced theoretically based on the Bessel function	Based on the phase modulation theory of BLODT, a test bed was developed to simulate single-point defects by generating torque vibration.	Xinbo Wu et al., 2015 [47]

**Table 4:** Summary and comparison table of artificial intelligence signal analysis methods.

Purpose	Applied Environment	Fault Types	Signal Processing Method	Based Model or Theory	Reference
Fault detection	At different load levels	Rotor rod fracture and bearing failure			Soualhi, A et al., 2013 [49]
Mechanical fault detection	Variable speed conditions	Rotor eccentricity and bearing fault detection of direct drive wind turbine	A high-resolution broadband synchronous sampling algorithm with high computational efficiency	High-resolution broadband synchronous sampling algorithm with high computational efficiency (a pulse detection algorithm)	Gong, Xiang et al., 2015 [50]
Fault diagnosis	Under different load conditions	Outer ring fault	The Fast Fourier transform		Shrinathan et al., 2019 [51]
	Noise environment	Bearing fault	Driving algorithm and parallel resonant controller and proportional-integral controller	Improved drive algorithm and Envelope spectrum analysis	Ming Yang et al., 2020 [56]
	Three-phase induction motors		The time domain fault feature extraction method is based on mutual information measurement between two phase current signals	Artificial neural networks, especially multi-layer perceptron, K-nearest neighbor, and support vector machine	Tiago et al., 2020 [57]
Rolling bearing fault diagnosis		External bearings in a rotary system	Fault diagnosis method based on Deep Learning and Information Fusion (IF) based on Motor CS		Hoang et al. 2020 [52]
	Noise environment	Multiple separation and combination failures	Deep-sincnet’s new DL architecture	Deep-sincnet’s new DL architecture	Abid, Firas Ben et al. 2020 [53]
		External bearing failure in driveline	Line spectrum feature extraction (LSFE) technology	Signal-based Adaptive Semi-supervised Framework (C-ASSF)	Jie Li et al., 2021 [54]
		Different types of bearing failures and load anomalies	Pre-trained Deep Convolutional Neural Networks (modified by transfer learning techniques)	Novel program	Marcello et al., 2021 [55]

**Table 5:** Summary and comparison table of time-frequency signal analysis methods.

Purpose	Applied Environment	Fault Types	Signal Processing Method	Based Model or Theory	Reference
Fault diagnosis	Stationary and non-stationary situations		Integrated Empirical Mode Decomposition (EEMD)		Amirat, Y et al., 2012 [59]
		The outer ring of the bearing is faulty	Continuous wavelet transform		Singh et al., 2017 [67]
			The wavelet packet transform classification method develops a deep one-dimensional convolutional neural network with a Softmax layer.		Kao, IH et al., 2019 [73]
			Adaptive neural fuzzy inference system (ANFIS)	Improved ANFIS model	Soualhi, Moncef et al., 2019 [74]
		Single point damage (bearing outer ring damage and bearing ball damage) and distributed damage (corrosion damage)			Victor Avina-Corral et al., 2021 [76]
Bearing fault condition monitoring			Short-time Fourier transform or discrete wavelet transform.	A diagnostic index based on residual subtraction energy is proposed.	ElBouchikhi, E et al., 2013 [60]
Rolling bearing fault detection and diagnosis			Current spectrum analysis	A novel parameterized spectrum estimator that makes full use of fault-sensitive frequencies	El Bouchikhi et al., 2015 [61]
		Defects in rolling bearing and rotor rod	A central kurtosis indicator is used to extract fault features from the spectrum of early IM defect detection in current signals	Center kurtosis indicator	Li, De Z. et al., 2015 [62]
			Algorithm of spectral kurtosis (fast kurtosis diagram and wavelet peak-to-peak diagram)		Leite, 2015 [63]
			Linear discriminant analysis evaluated the characteristics		Mboo et al., 2016 [64]
		Bearing outer raceway fault			Song, XJ et al., 2017 [65]
	Noise condition	Defects in outer raceway, inner raceway and ball bearings	Spectrum analysis		Dalvand, Fardin et al., 2017 [66]
					Dalvand, Fardin et al., 2018 [68]
			Motor current characteristic analysis (MCSA), discrete wavelet transform (DWT), steady wavelet transform (SWT), and wavelet packet decomposition (WPD) were used for spectrum subtraction		Kompella et al., 2018 [69]
			Hilbert transform, Fast Fourier transform (FFT) spectrum analysis		Song, XJ et al., 2018 [70]
		Outer ring peeling, inner ring peeling and ball peeling	Iterative numerical integration method	Stator current model	Han, Qinkai et al., 2019 [72]
			Modified Winding Function Method (MWFA) Electromechanical magnetic coupling calculation model for induction Motor (IM)	Electromechanical Magnetic Coupling Calculation Model for Induction Motor (IM) Based on Improved Winding Function Method (MWFA), New Evaluation Index FHD (Fault Excitation Harmonic Distortion)	Wang, Chen et al., 2021 [75]
Feature extraction and fault classification	Under various bearing conditions and load levels		Optimizing BFD mixing method for static wavelet packet transform		Ben Abid, ETAL., 2018 [71]
Bearing characteristic measurement	Self-aligning ball bearings		Analysis of vibration spectra	recursive quantification analysis (RQA)	Bartłomiej Ambrożkiewicz et al., 2022 [77]

**Table 6:** Summary and comparison table of spectral signal analysis methods.

Purpose	Fault Types	Signal Processing Method	Based Model or Theory [81]	Reference
Fault diagnosis		Fault feature frequency and amplitude estimator	Multi-dimensional MUSIC (MD MUSIC) algorithm, fault feature frequency amplitude estimator	Elbouchikhi et al., 2016 [77]
	Bearing and rotor rod fracture failure	Frequency estimation techniques and least square estimators	State monitoring architecture based on stator current measurement	Trachi, Y et al., 2016 [78]
		Using advanced signal processing tools	Matrix pencil method and wavelet de-noising	K. C. Deekshit Kompella et al., 2020 [79]
Condition monitoring		Installing an acceleration sensor used for state detection inside a bearing	Divide the measured acceleration into the angular and linear components	Vladimir V Sinitsin et al., 2017 [80]

**Table 7:** Summary and comparison table of remote real-time monitoring methods.

Purpose	Fault Types	Signal Processing Method	Based Model or Theory	Reference
Bearing condition detection	Insufficient lubrication		Torque model	Jezic Von Gesseneck, J. et al., 2016 [81].
Fault diagnosis		Fast Fourier Transform (FFT), Independent Element Analysis (ICA) method		Yang, T et al., 2016 [82]
Online status monitoring		Operate the test stand	Test bed	Corne, B et al., 2018 [83]
Fault diagnosis and detection		PFA upper limit and average Detection delay (ADD) form	New multi-candidate low-delay detection algorithm	Samrat Nath et al., 2020 [84]
Bearing fault diagnosis	The inner raceway is faulty	The Fast Fourier transform	Multi-coupling circuit modeling	Ojaghi, M. et al., 2018 [85]

**Table 8:** Summary and comparison table of other methods based on one-dimensional signals.

Purpose	Applied Environment	Fault Signal	Fault Types	Signal Processing Method	Based Model or Theory	Reference
Rolling bearing fault diagnosis		Acoustic emission signal		Multiscale morphological analysis program		Wen-Jing et al., 2016 [86]
Rolling bearing fault detection and diagnosis		Speed signal		Principal Component Analysis (PCA) of absolute value	A novel BFD technique	Hamadache et al., 2015 [87]
	Noise environment			Kurtosis spectrum analysis	Integrated model of motor and bearing	Boyang et al., 2019 [88]
Fault diagnosis		Velocity signal & residual signal	Bearing fault	Discrete Wavelet Transform (DWT) and Continuous Wavelet Transform (CWT)		Omid Zandi et al., 2019 [89]
Bearing fault diagnosis		The magnetic field frequency		Multimodulated torque wave frequencies, power harmonics, and slot harmonics, finite element analysis	Magnetic field characteristic frequency expression	Tianyu Geng et al., 2016 [90]
Bearing fault diagnosis		Neutral voltage	Bearing wear and loosening	Euler-Lagrange equations for induction motors, Spectral analysis of neutral voltages	A new comprehensive modeling and analysis method for 3-DOF induction motor and an effective bearing looseness characteristic method is proposed	Mohammad J. Jafarian et al., 2020 [91]
Bearing fault diagnosis	No load and full load conditions	Instantaneous power				Irfan, M et al., 2015 [92]
Fault diagnosis of rolling bearings	Noise condition	The pulse signal		Spectral correlation analysis in the frequency domain, adaptive algorithm based on peak energy	Scale selection method for multiscale mathematical morphology	Qiang et al., 2021 [93]
Early fault diagnosis of rolling bearings		Component of the signal	Early failures of rolling bearings	Singular value decomposition, inverse transformation		Leng et al., 2014 [94]
Fault diagnosis of rotating machinery	Noise condition	Encoder signal		A rotary encoder is a systematic framework for the health assessment of rotary machinery.	A system framework for health assessment of rotating machinery using a rotary encoder is established	Zhao, Ming et al., 2018 [95]

**Table 9:** Summary table of two-dimensional-image methods.

Purpose	Fault Signal	Signal Processing Method	Imaging Method	Proposed Model	Reference
Motor rolling bearing fault diagnosis	Vibration image	Short Time Fourier Transform (STFT)	A time-frequency representation vibration image is obtained from the raw data	Adapted Image Classification Transformer (ICT) model (alternative to CNN)	Christos T. Alexakos et al., 2021 [96]
Bearing fault diagnosis		Using persistent spectrum	Using persistent spectrum		Mohamed et al., 2022 [98]
The rolling bearing vibration image samples affected by intense noise were studied.		CNN	IMFA, EP, SPCI, GTM	CNN adaptive feature extraction and recognition model	Fan, Hongwei et al., 2021 [99]
Rolling bearing fault diagnosis		The Fast Fourier transform		Linear model	Bin Liu et al., 2021 [97]
		DA-RNN model	The vibration signal is converted into an image by the vibration acceleration signal, and the corresponding integral velocity and displacement signal.	Rolling bearing fault diagnosis model combining two-stage attention recursive neural network (DA-RNN) and convolution block attention module (CBAM)	Jun Li et al., 2022 [100]
			LBP conversion	Wgan-gp data extension method	Hongwei Fan et al., 2022 [101]
		DenseNet	GAF		JIANG Jiaguo et al., 2021 [102]
		Adam small batch optimization method	Gram Angle difference field	An intelligent fault diagnosis model based on Gram Angle Difference field (GADF) and improved convolutional Neural network (CNN)	School of Mechanical and Electrical Engineering, 2021 [103]

**Table 10:** Summary and comparison table of grayscale image methods.

Purpose	Fault Signal	Signal Processing Method	Proposed Model	Reference
Rolling bearing fault diagnosis	Gray image	RGBVI-CNN	Rgbvi-cnn classification model	Hosameldin O. A. Ahmed et al., 2021 [104]
		The pre-trained off-CNN initializes the on-CNN parameters with the off-CNN parameters	Rolling bearing fault diagnosis model based on online Transfer Convolutional Neural Network (OTCNN)	Quansheng Xu et al., 2022 [106]
		GAN		Hongtao Tang et al., 2021 [107]
Fault locations of bearing components are classified		Wave atomic transformation	Multi-class support Vector Machine (MSVM) model	Jha, Rakesh Kumar et al., 2021 [105]
Accurate, intelligent fault diagnosis of rolling bearings is realized		Convolved with different convolved kernels	Based on MCNN and data fusion model	Lv, Defeng et al., 2021 [108]

**Table 11:** Summary and comparison table of SDP image methods.

Purpose	Fault Signal	Signal Processing Method	Imaging Method	Proposed Model	Reference
Bearing fault diagnosis	SDP image (using symmetric lattice principle)	Incremental accumulation	Based on SDP method		Xuewei Song et al., 2022 [109]
		Symmetric lattice (SDP) principle	Symmetric lattice (SDP) principle	Optimal Convolutional Neural Network (CNN) model	Yongjian Sun et al., 2022 [110]
		Normalized processing	Empirical Mode Decomposition (EMD)	Improved Chebyshev Distance (a New Characteristic Index)	Sun, Yongjian, 2021 [111]

**Table 12:** Summary and comparison table of other types of vibration image methods.

Purpose	Fault Signal	Signal Processing Method	Imaging Method	Proposed Model	Reference
Feature classification of rolling bearing fault reconstruction	Two-dimensional time series images	The Summation field at Cape Gram is rebuilt	The original vibration signals collected were reconstructed using gram Angle summation field	RGBVI-CNN	Yu, Hu et al., 2022 [112]
Bearing fault diagnosis	Composite color image	Fuses multi-domain information	the time-domain signal, and the envelope spectrum of time-frequency analysis	An autonomous diagnostic system based on image conversion technology and Neural convolutional network (CNN) assisted multi-task learning (MTL).	Hasan et al., 2022 [113]
The improved traditional time-frequency analysis technique is difficult to be applied to rolling bearing vibration signal (non-stationary signal)	Snowflake image (symmetric image)	Empirical Mode Decomposition (EMD)	Empirical Mode Decomposition (EMD)	Improved Manhattan Distance (new feature)	Sun, Yongjian, 2021 [114]
Rolling bearing fault diagnosis		CNN model	Softmax classifier	CNN model	Wang, H, 2019 [115]
Bearing fault diagnosis	Multi-domain image	Multi-branched structure of DNN		Multi-branched structure of DNN	Van-Cuong Nguyen et al., 2021 [116]
It plays a decisive role in the safe operation of the whole unit. Aiming at the rolling bearing fault diagnosis problem of unit	Recursive figure (RP)	According to the visual invariance and acceleration robustness of the human visual system (HVS)			Yujie Cheng et al., 2017 [117]
Rolling bearing fault diagnosis	Order time figure	CNN model		CNN model	Tayyab et al., 2022 [118]
Fault diagnosis		MTF-CNN	Time series are converted into images	MTF-CNN	Mengjiao Wang et al., 2022 [119]

**Table 13:** Summary and comparison table of other two-dimensional-image methods.

Purpose	Fault Signal	Signal Processing Method	Imaging Method	Proposed Model	Reference
Mitigate the shortcomings of existing ABD methods	Acoustic imaging	Microphone array acquisition	Wave superposition method(WSM)		Ran Wang et al., 2020 [120]
Bearing faults are classified	Spectral imaging	Normalized amplitude conversion	Spectral content normalized amplitude is converted into an image		Youcef Khodja et al., 2020 [121]
Rolling bearing fault diagnosis		Principle of invariant moment	Moment invariant S transformation		Guo, JF et al., 2013 [122]
		Deep CNN uses transfer learning		Stochastic gradient descent (SGD), adaptive moment estimation (Adam) and Adamax models	Chhaya Grover et al., 2022 [123]
Rolling bearing fault diagnosis	Infrared thermal image	Artificial Neural Network (ANN) and CNN based on Lenet-5 structure		CNN based on Lenet-5 structure	Choudhary et al., 2021 [124]

**Table 14:** Summary and comparison table of time-frequency image methods.

Purpose	Fault Signal	Signal Processing Method	Imaging Method	Proposed Model	Reference
Rolling bearing fault diagnosis	The time -frequency image	Binary processing, multiplication operation			Ma, Yunchao et al., 2021 [125]
			EMD-PWVD	FCM clustering of vibration images	Hongwei Fan et al., 2020 [126]
		Position constrained linear coding (LLC) and spatial pyramid matching methods			Wei Gang Wang et al., 2014 [133]
		Extraction method of ridge line	Using ridge extraction method, resonance demodulation, scaling instantaneous frequency of frequency axis		Wang, Y et al., 2019 [135]
		SPWVD time-frequency analysis method	SPWVD time-frequency analysis method		Yang Hongbai et al., 2017 [136]
		Classification and identification based on UCI datasets	An algorithm for solving trace ratio problems in extended linear discriminant analysis	Construct feature data sets	Zhao, MB et al., 2014 [137]
		Deep convolutional generative adversarial network expansion	Continuous wavelet transform		Tian Han et al., 2021 [140]
Bearing fault diagnosis				The universal Alexnet bearing fault diagnosis model is replaced on the fully connected layer	Wang, JY et al., 2019 [127]
Rolling bearing fault severity monitoring		Sparse time-frequency analysis based on first-order Primal-dual algorithm (STFA-PD)	Sparse time-frequency analysis method based on first-order Primal-dual algorithm (STFA-PD)	STFA-PD model	Du, Y et al., 2018 [128]
Detect bearing failure types		Linear prediction theory with optimal prediction order	Kurtosis and spectral analysis		Yuan, HD et al., 2018 [129]
CNN system is used to classify and diagnose rolling bearing faults		Three time-frequency analysis methods (SHORT-time Fourier transform, Wavelet transform and Hilbert-Huang transform)	Time-frequency representation generates image representation	CNN model	David Verstraete et al., 2017 [130]
In order to improve the accuracy of rolling bearing fault diagnosis		SPWVD time-frequency analysis method	SPWVD time-frequency analysis method was used		Wang, Yaping et al., 2017 [131]
In order to overcome the shortcoming of the fast kurtosis method, a method to remove noise in the whole frequency band is needed.		Weak signal extraction method based on time-frequency distribution image dimension reduction			Wang, Y et al., 2016 [132]
Rolling bearing pattern recognition		Hough transform for wigner-Ville time-frequency distribution			Li, Hongkun et al., 2010 [134]
Bearing fault diagnosis		Smooth Global Gradient Class Activation Map (SGG-CAM), EMSCNN Network, Frequency Slice Wavelet Transform (FSWT)	Slice wavelet transform (FSWT)	Efficient multiscale convolutional neural networks (EMSCNN)	Hongchun Sun et al., 2022 [138]
		Short time Fourier transform, deep residual network	Short time Fourier transform	Multi-sensory data Fusion method based on deep residual network	Gultekin, Ozgur et al., 2022 [139]

## Data Availability

Not applicable.
